# Amaranth Oil for Dermatologic Conditions: Inflammation Control and Cytotoxicity Assessment in Skin-Related Cell Models—Preliminary Study

**DOI:** 10.3390/molecules31060968

**Published:** 2026-03-13

**Authors:** Paweł Paśko, Agnieszka Galanty, Ewelina Prochownik, Alma Leticia Martinez-Ayala, Alma Chu-Martínez, Pitipong Thobunluepop, Danail Pavlov, Aviva Friedman-Ezra, Shela Gorinstein

**Affiliations:** 1 Department of Food Chemistry and Nutrition, Jagiellonian University Medical College, 30-688 Kraków, Poland; ewelina.gajdzik@uj.edu.pl; 2 Department of Pharmacognosy, Jagiellonian University Medical College, 30-688 Kraków, Poland; agnieszka.galanty@uj.edu.pl; 3 Centro de Desarrollo de Productos Bióticos, Instituto Politécnico Nacional, Yautepec 62731, Morelos, Mexico; alayala@ipn.mx (A.L.M.-A.); achum1200@alumno.ipn.mx (A.C.-M.); 4 Department of Agronomy, Faculty of Agriculture, Kasetsart University, Chatuchak, Bangkok 10900, Thailand; fagrppt@ku.ac.th; 5 Laboratory of Nutrigenomics, Functional Foods and Nutraceuticals, Department of Biochemistry, Molecular Medicine and Nutrigenomics, Faculty of Pharmacy, Medical University of Varna, 9002 Varna, Bulgaria; danailpavlov@gmail.com; 6 Institute for Drug Research, School of Pharmacy, Faculty of Medicine, The Hebrew University of Jerusalem, Jerusalem 9112001, Israel; aviva.friedman-ezra@mail.huji.ac.il

**Keywords:** amaranth oil, skin, topical use, cytotoxicity, skin cancer, inflammation, human serum albumin binding, food for skin, rose oil

## Abstract

Amaranth oil (AMO) and its topical formulation enriched with rose oil (AMOR) were evaluated for anti-inflammatory and cytotoxic properties in skin-relevant models. Two complementary inflammation models were used to assess immunomodulatory potential, (i) LPS-stimulated macrophages and (ii) TNF-α/IFN-γ-stimulated immortalized HaCaT keratinocytes, while cytotoxicity and selectivity were tested on human HaCaT keratinocytes and melanoma cell lines (A375, HTB140). GC-MS and FTIR analyses were performed to confirm the presence of key bioactive compounds (squalene, fatty acids, phenolics). AMOR showed significantly higher polyphenol and palmitic acid content than AMO. In both inflammation models, AMOR more effectively reduced IL-6, IL-1β, and TNF-α release. Cytotoxicity assays revealed that both oils were safe for normal keratinocytes, while selectively cytotoxic to melanoma cells, with AMOR demonstrating greater potency (IC_50_ A375 = 3.8 μg/mL and HTB140 = 18.9 μg/mL). Albumin-binding studies showed that AMOR had stronger interactions with these proteins, which may enhance delivery and tissue retention. In conclusion, both oils exhibit promising topical safety, but AMOR provides enhanced anti-inflammatory and cytotoxic effects due to its enriched composition. This study supports the therapeutic potential of amaranth oil in different skin diseases, especially when combined with essential oils of complementary bioactivity.

## 1. Introduction

Amaranth seeds and their by-products such as oil have been gaining increasing scientific and medical interest in recent years. Traditionally valued as a component of functional foods, particularly for cardiovascular health and as a potential chemopreventive agent in cancers such as prostate cancer [1,2], they are now also becoming recognized for their dermatological applications. Due to its unique lipid profile, characterized by high levels of linoleic and oleic acids, tocopherols, sterols, and exceptionally abundant squalene, amaranth oil can be considered as a promising topical agent, supporting skin health. However, the literature data in this area is scarce, indicating a significant gap needing to be filled.

The existing evidence suggests that amaranth seed oil may be beneficial in a range of skin conditions. According to traditional and empirical reports, amaranth oil has been used topically in some inflammatory and irritation-related skin conditions, including psoriasis, eczema, atopic dermatitis, neurodermatitis, dry seborrhea, acne, furunculosis, and herpetic skin eruptions. However, it should be emphasized that most of these claims originate from non-scientific sources, such as traditional medicine websites and naturopathic practice reports, and are not yet sufficiently supported by controlled clinical studies. These uses are primarily based on the anti-inflammatory, soothing, and barrier-supporting effects of amaranth seed oil [3]. A study on human dermal fibroblasts demonstrated that amaranth seed oil counteracts UVA-induced inhibition of collagen production, normalizes prolinase activity, suppresses inflammatory markers such as NF-κB and COX-2, and accelerates wound healing in vitro. These findings support the potential use of amaranth oil in post-sun exposure care and the prevention of photoaging, which is probably attributed to its high squalene content [4]. Amaranth oil has been evaluated in a rat model of thermal burn injury, where its topical application led to improved wound cleansing, the removal of necrotic tissue, enhanced granulation and re-epithelialization, and normalization of oxidative enzyme activity [3]. Experimental data additionally show that amaranth oil enhances fibroblast migration in the scratch-wound model, and partially restores collagen biosynthesis suppressed by UVA irradiation [5].

Commercially, amaranth oil is available mostly as pure cold-pressed seed oil. Moreover, amaranth oil has been used to prepare W/O formulations, based on which an effective moisturizing cream was produced [6].

The growing interest in amaranth oil within dermatology is closely associated with its favorable fatty acid composition and bioactive lipid profile mentioned earlier. Amaranth oil is particularly rich in linoleic acid and oleic acid (approximately 40 and 22%, respectively) and contains an exceptionally high amount of squalene, among the highest reported for edible plant oils. In addition, it provides significant amounts of tocopherols and phytosterols. This unique composition underlies its antioxidant, regenerative, and anti-inflammatory properties, making amaranth oil a promising candidate for topical application in various skin conditions [7].

Traditional and empirical reports suggesting topical use of amaranth oil in various inflammatory and irritation-related skin disorders and the lack of systematic scientific studies addressing these issues inspired us to design the present study. We aimed to verify the effects of pure amaranth oil (AMO) and commercially available amaranth oil formulation with rose oil (AMOR) in terms of their impact on the viability of different human skin normal and cancer cells. These analyses were complemented by the assessment of anti-inflammatory activity of the oils, using two independent in vitro models: human immortalized keratinocytes (HaCaT) stimulated with a cytokine cocktail and murine macrophages (RAW 264.7 cells) stimulated with lipopolysaccharide (LPS). The in vitro biological studies were preceded by a detailed chemical characterization of the oils, including the analysis of fatty acid profiles, squalene content, total polyphenolic compounds and FTIR to confirm the presence of rose oil additives. Finally, as serum albumin is the principal transport protein for lipophilic bioactive compounds in extracellular fluids, evaluation of the human serum albumin (HSA) binding properties of the oils was performed to provide deeper insight into the biological behavior of oil-derived antioxidants and formulation components. Such interactions may influence tissue distribution, local retention, and potential biological effects relevant to skin disorders.

## 2. Results and Discussion

### 2.1. Chemical Composition of the Evaluated Amaranth Oils

In the comparison between the amaranth oil (AMO) and amaranth oil with rose oil (AMOR) compositions (Table 1), significantly higher levels of palmitic acid were observed in the AMOR samples, suggesting compositional changes due to the refinement or enrichment. Differences in predominant fatty acids, including stearic, oleic, and linoleic acid content, were not statistically significant. Linoleic, oleic, palmitic and stearic acids were also identified as the predominant fatty acids in amaranth oils obtained from different species of this plant by Azri et al. [8]. Additionally, these data are in accordance with our previous study on different pseudocereal oils [2].

AMOR showed a significantly higher total polyphenol content compared to AMO, confirming effective enrichment by rose oil. Both the lipophilic and hydrophilic fractions were also markedly elevated in AMOR, indicating enhanced antioxidant potential across both polar and non-polar compounds. Amaranth is recognized as one of the richest plant-based sources of squalene. Our results showed squalene content ranging from 6.9 to 7.9%, which aligns with values reported by other authors ranging from 2.4 to 8.0% [8,9], and even up to 12% in wild amaranth varieties [10]. Although AMOR contained slightly higher levels of squalene than AMO, the difference was not statistically significant.

### 2.2. FTIR of Amaranth Oils

We used FTIR analysis to confirm two key aspects: (1) the presence of active compounds such as squalene and phenolics and (2) the addition of rose oil through detection of its characteristic functional groups. Additionally, FTIR spectra were used to verify if the enrichment with rose oil did not alter the amaranth oil’s fundamental chemical composition.

**Figure 1:**
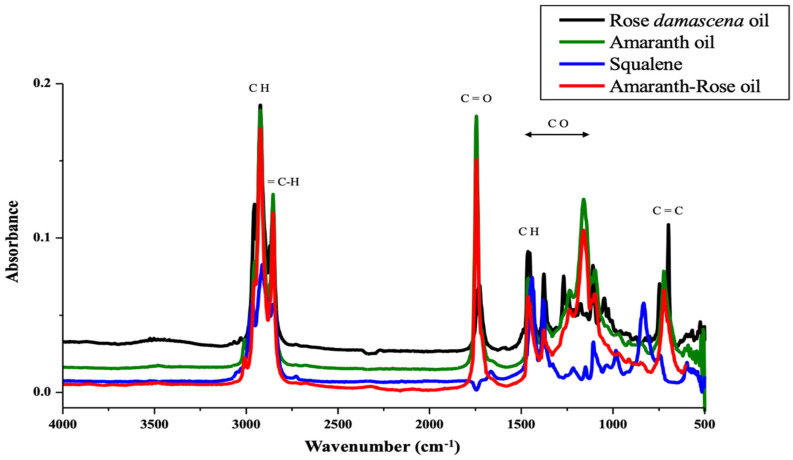
FTIR spectra of amaranth oils combined with rose oils and squalene as reference material. For each point, 3 samples were analyzed and are shown in the figure. *y*-axis = absorbance, *x*-axis = wavelength (cm^−1^).

The FTIR spectra of rose oil, amaranth oil, their combined formulation, and squalene exhibit several characteristic absorption bands (Figure 1). The band observed at 3008 cm^−1^ is characteristic of the stretching vibration of olefinic =C-H bonds, present in vegetable oils rich in unsaturated fatty acids [2]. The bands near 2954 cm^−1^, 2924 cm^−1^, and 2853 cm^−1^ correspond to the asymmetric stretching vibrations of C-H bonds in methylene (CH_2_) groups. These bands are present in all the analyzed samples, indicating the presence of long hydrocarbon chains, common in vegetable oils and squalene.

The band at 1746 cm^−1^ corresponds to the carbonyl (C=O) stretching in triglycerides and is observed in amaranth oil, rose oil, and the amaranth–rose formulation, indicating the presence of triglyceride esters as components of these oils. In the absorption region of 1654–1300 cm^−1^, bands related to C-H bond bending vibrations are observed, reflecting the presence of aliphatic chains. This band is relevant to squalene, as described in FTIR studies focused on squalene characterization. The bands observed at 1462 cm^−1^ are attributed to the bending vibrations of the aliphatic CH_2_ and CH_3_ groups, while the band at 1377 cm^−1^ corresponds to the symmetric bending of the CH_3_ groups. The spectral region between 1178 cm^−1^ and 1036 cm^−1^ is associated with C-O stretching vibrations of ester groups. Finally, the band observed at 646 cm^−1^ corresponds to out-of-plane bending vibrations of the cis-HC=CH group present in unsaturated fatty acids [11]. Some of the observed absorption bands can be attributed to squalene, particularly the regions associated with aliphatic C-H stretching at 2954–2853 cm^−1^, cis C=C stretching at 1654 cm^−1^ and the bending of methylene and methyl groups at 1462 cm^−1^ [2,12]. Overall, all analyzed samples shared common absorption bands at 2954–2924 and 2853 cm^−1^, corresponding to asymmetric and symmetric C-H stretching vibrations of the CH_3_ and CH_2_ groups, as well as a band around 1654 cm^−1^ attributed to cis C=C stretching. Additionally, a CH_2_CH_3_ bending band was observed at 1462 cm^−1^ in all spectra. The combined amaranth–rose oil formulation exhibits a spectral profile that integrates structural characteristics of both oils. Amaranth oil is recognized for its content of unsaturated fatty acids and squalene, compounds associated with antioxidant, anti-inflammatory, and regenerative properties [2]. Furthermore, rose oil contains terpenes and aromatic alcohols, widely documented for their antioxidant, antimicrobial, and healing activity [13,14].

### 2.3. Cytotoxicity of Amaranth Oil Towards Human Skin Cells

The cytotoxic effects of amaranth oil (AMO) and amaranth oil with rose oil (AMOR) were evaluated over a wide concentration range (2.5–100 µg/mL) in human skin cells, including two melanoma cell lines (HTB140 and A375) differing in metastatic potential, as well as in non-cancerous immortalized keratinocytes (HaCaT).

A strong cytotoxic effect of both examined oils was observed in melanoma cell lines. The viability of highly metastatic HTB140 cells exposed to AMO was reduced to 22.7 ± 1.8 and 7.4 ± 2.5% at the two highest tested doses of 50 and 100 µg/mL, respectively. The AMOR formulation exhibited an even stronger effect, reducing the viability to 0–0.5%, indicating a near-complete loss of viable tumor cells. Similarly, malignant A375 melanoma cells were highly sensitive to this treatment. AMO reduced cell viability to 5.5 ± 1.0 and 0.5 ± 0.2% at concentrations of 50 and 100 µg/mL, respectively, whereas AMOR reduced viability completely at both concentrations, demonstrating strong, maximal cytotoxic activity. In contrast, both oils showed only weak toxicity toward immortalized HaCaT keratinocytes, with high cell viability maintained across the tested concentrations, confirming their safety profile for potential topical application. At the highest tested concentration, HaCaT viability reached 90.5 ± 3.0% for AMO and 87.2 ± 2.2% for AMOR, indicating good tolerability of both formulations in these cells.

Overall, both oils exhibited selective cytotoxicity toward melanoma cells with minimal toxicity to immortalized, non-cancerous keratinocytes. The AMOR formulation showed stronger cytotoxic activity than amaranth oil alone, which was further confirmed by IC_50_ values (Table 2).

Our previous study indicated that melanoma cells were the most sensitive to various pseudocereal oils, including amaranth, different types of quinoas, and buckwheat oils. Within the skin cancer panel, malignant A375 cells were sensitive to all tested oils, with IC_50_ values for buckwheat and yellow quinoa oil as low as 5.6 and 6.3 µg/mL, respectively [2]. In the present study, a new formulation of amaranth oil supplemented with rose oil demonstrated even greater cytotoxicity toward these cancer cells (IC_50_ = 3.8 µg/mL) compared with standard amaranth oil alone.

Paśko et al. [2] also reported that in a more metastatic HTB140 cell line, the most promising cytotoxic activity was observed for yellow quinoa oil (IC_50_ = 10.4 µg/mL). Wolosik et al. [5] showed that application of *A. cruentus* oil to UVA-treated human dermal fibroblasts (0.1 and 0.15%) reduced the expression of several apoptosis markers (p53, caspase-3, caspase-9, and PARP). Additionally, this oil restored the expression of p-Akt and mTOR proteins, which are associated with antioxidant responses via Nrf2 pathway activation. Lacatusu et al. [15] predicted that the enhanced antioxidant and photoprotective properties of sunscreen formulations based on nanostructured lipid carriers containing amaranth oil may reduce skin cancer risk and delay photoaging effects. Chauhan et al. [16] indicated that amaranth oil, due to its high squalene content, may be beneficial in skin cancer, showing strong predicted binding to key melanoma signaling proteins such as BRAF and TGF-β, suggesting potential inhibition of tumor growth pathways. Moreover, squalene demonstrates antioxidant and membrane-protective properties, which may reduce oxidative stress and modulate cancer-related signaling, supporting its potential chemopreventive and adjunct therapeutic role. This potential may be further enhanced by adding rose oil to topical formulations, as was also proved by our results. Rose oil has demonstrated photoprotective and anti-inflammatory effects in the skin, including reduction in UVB-induced oxidative stress, increased antioxidant enzyme activity (CAT, SOD), decreased inflammatory mediators, and downregulation of the NF-κB and MAPK signaling pathways. These mechanisms are directly relevant to skin carcinogenesis and photo-damage [17]. However, the combined mechanism of action of amaranth oil and rose oil in skin cancer prevention requires further detailed mechanistic and in vivo investigation.

### 2.4. Anti-Inflammatory Activity of Amaranth Oils

To obtain a more comprehensive assessment of the anti-inflammatory potential of amaranth oil formulations, we employed two complementary in vitro inflammation models: LPS-stimulated RAW 264.7 murine macrophages and cytokine-stimulated human immortalized keratinocytes (HaCaT). This dual approach was chosen because the current evidence for the anti-inflammatory effects of amaranth oil, especially in skin-relevant models, remains limited and not fully convincing, and inflammatory processes in the skin are driven by multiple interacting cell types. The RAW 264.7 model primarily reflects innate immune activation and systemic inflammatory signaling, where macrophages represent a major source of TNF-α, IL-6, and nitric oxide [18]. Therefore, the results from this model may approximate the anti-inflammatory potential of the formulations under conditions resembling internal exposure (e.g., after oral intake and systemic distribution of bioactive compounds of amaranth oil). In contrast, the HaCaT inflammation model is frequently used in screening studies of substances with possible inhibitory effects on the production of proinflammatory mediators, involved in skin inflammation. As immortalized HaCaT cells do not fully reflect physiological epidermal inflammatory responses, compared to primary keratinocytes, the model is obviously simplified, but still can provide reliable results [19,20] to be further tested in more accurate skin-targeted models.

Using both models, especially in the case of AMOR, which is dedicated only for external use, allows us to evaluate whether the oils modulate inflammation at two critical levels: immune-cell-driven inflammation and skin barrier/epidermal inflammation, thereby improving the translational relevance of the findings. This is particularly important because amaranth oil could potentially be used as supportive therapy in skin disorders via both topical and internal routes, and consistent anti-inflammatory effects across these models strengthen the rationale for further mechanistic and in vivo studies.

#### 2.4.1. Anti-Inflammatory Activity of Amaranth Oils in Macrophage Model

**Figure 2:**
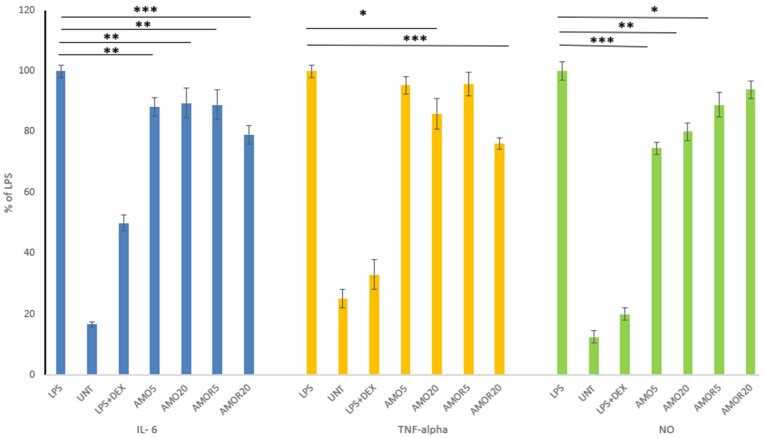
Effect of amaranth oil (AMO) and rose-enriched amaranth oil (AMOR) at two concentrations of 5 and 20 ug/mL (5) (20), and dexamethasone as reference drug (DEX), on LPS-induced inflammatory mediator production (IL-6, TNF-α, and nitric oxide) in stimulated (LPS) cells. Untreated cells (UNT) were also included. Data are expressed as % of LPS control (mean ± SD, *n* = 3). Statistical significance was evaluated using one-way ANOVA with post hoc testing (* *p* < 0.05, ** *p* < 0.01, *** *p* < 0.001 vs. LPS).

The anti-inflammatory effects of amaranth oils (AMO and AMOR) were evaluated in an LPS-stimulated macrophage model by measuring IL-6, TNF-α, and nitric oxide production (Figure 2). Both AMO and AMOR at the tested concentrations significantly reduced IL-6 levels compared with LPS-stimulated controls. No significant dose-dependent difference was observed for AMO, whereas AMOR produced a significantly stronger reduction at a concentration of 20 (AMOR20) compared with 5 (AMOR5) (*p* < 0.05). Between both formulations, no difference was detected at the lower dose, while at the higher dose, AMOR showed greater IL-6 suppression than AMO (*p* < 0.05).

Compared with LPS-stimulated cells, a significant reduction in TNF-α release was observed for a higher dose of oil (20) (AMO20 (*p* < 0.05) and AMOR20 (*p* < 0.001)), whereas a lower dose of both oils showed only weak inhibitory potential. Dose-dependent differences were significant within both formulations (AMO5 vs. AMO20, *p* < 0.05; AMOR5 vs. AMOR20, *p* < 0.01). Between the formulations, no difference was observed at the lower dose, whereas at the higher dose, AMOR showed significantly stronger inhibition than AMO (*p* < 0.01). Compared with LPS control, significant reductions in nitric oxide production were observed for AMO5 (*p* < 0.001), AMO20 (*p* < 0.01), and AMOR5 (*p* < 0.05), while AMOR20 showed no significant effect. Differences between the formulations were significant at both tested doses (AMO vs. AMOR: *p* < 0.05), whereas dose-dependent differences within each formulation did not reach statistical significance.

Generally, the inhibitory effects were most pronounced for TNF-α and NO in the macrophage model, which are key mediators of inflammatory signaling and oxidative-stress-associated tissue damage relevant to skin pathology and carcinogenesis [21,22].

Across all measured markers, the rose-enriched formulation (AMOR) showed consistently greater inhibitory activity than pure amaranth oil, although the magnitude of the difference was moderate. This enhanced activity, related to the addition of rose oil, is mechanistically plausible and aligns with previously reported properties of its components, including antioxidant, photoprotective, and NF-κB/MAPK pathway-modulating effects. Geraniol, citronellol and nerol might act as possible binding partners of MAPKP38α, which can modulate their functional activity via decreasing the accessibility of substrate. Geraniol may have a high potential to inhibit their function [23]. These pathways are directly involved in inflammatory signaling and skin damage progression [24].

Our previous study [2] demonstrated that pseudocereal oils (amaranth, quinoa, buckwheat) differed in their anti-inflammatory effects in LPS-stimulated macrophages. Significant NO inhibition was observed for most oils. Only quinoa (red and white) and buckwheat oils reduced TNF-α levels, without dose-dependent trends. All pseudocereal oils suppressed IL-6 release. These findings support the immunomodulatory potential of pseudocereal oils and complement our current data on AMO, highlighting its anti-inflammatory role via cytokine suppression.

#### 2.4.2. Anti-Inflammatory Activity of Amaranth Oils in HaCaT Model

**Figure 3:**
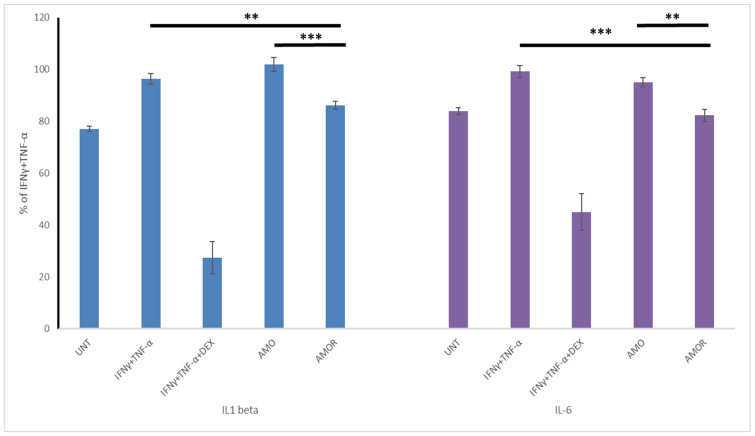
Effect of amaranth oil (AMO) and rose-enriched amaranth oil (AMOR), and dexamethasone as reference drug (DEX), on IL-1β and IL-6 levels in IFNγ+TNF-α-stimulated HaCaT model of inflammation expressed as a percentage of the IFNγ+TNF-α control group. Untreated cells (UNT) are also included. Data are presented as mean ± SD (*n* = 3) of three independent experiments. Statistical significance was evaluated using one-way ANOVA with post hoc testing (** *p* < 0.01, *** *p* < 0.001).

The second model of anti-inflammatory activity of amaranth oils in immortalized HaCaT cells showed that only AMOR significantly reduced the release of proinflammatory cytokines (Figure 3).

AMOR showed a pronounced effect (*p* < 0.01) in decreasing IL-1β levels in IFNγ+TNF-α-stimulated cells. For IL-6, AMOR treatment led to its statistically significant reduction (*p* <0.001). Direct comparison between AMO and AMOR revealed highly significant differences for both cytokines (*p* < 0.001 for IL-1β and *p* < 0.01 for IL-6), confirming the superior anti-inflammatory efficacy of AMOR in this model.

To strengthen the interpretation of our cytokine data, we extended our analysis by calculating the ratios of IL-6/IL-10 and IL-1β/IL-10, which are established indicators of the balance between pro- and anti-inflammatory responses. The results confirmed our previous findings: the IL-6/IL-10 and IL-1β/IL-10 ratios were lowest in the AMOR group (0.372 and 4.25, respectively) compared to AMO (0.490 and 6.11) and the inflammatory control IFNγ+TNF-α (0.447 and 6.24). These data indicate that AMOR promotes a more anti-inflammatory profile, likely due to its enriched composition and higher polyphenol content. Although most studies report IL-6, IL-1β, and IL-10 levels separately, the use of IL-6/IL-10 and IL-1β/IL-10 ratios provides a deeper insight into the inflammatory/anti-inflammatory balance. To our knowledge, such ratio-based analysis has not yet been widely applied in the context of edible plant oil research, making this approach a novel contribution to cytokine profiling in cell-based inflammation models and natural products’ impact [25].

### 2.5. Human Serum Albumin Binding Potential of Amaranth Oil Active Compounds

The interaction of bioactive oil components with human serum albumin (HSA) provides important information on their binding capacity, stability, and transport potential in biological systems. Our fluorescence binding analysis demonstrated that antioxidant-rich fractions, particularly polyphenolic compounds, can significantly modify HSA fluorescence intensity, indicating strong molecular interactions. Since amaranth oil contains bioactive antioxidant constituents, including squalene and phenolic compounds, their albumin-binding capacity may influence their bioavailability, distribution, and persistence in skin tissue after topical application. This aspect is especially important when different new formulations are applied in practice to the standard matrix, like additive plant essential oils rich in phenolic compounds, and when considering the use of these products in the prevention and treatment of different skin diseases [26]. From a practical perspective, albumin–ligand interactions are also relevant for external dermatological use, as albumin and albumin-like binding proteins present in interstitial fluids and wound exudates may act as local carriers and reservoirs for lipophilic antioxidant compounds. Such interactions may prolong local activity and enhance protective and anti-inflammatory effects in the skin microenvironment. Therefore, albumin was used not as a model of systemic transport, but as a representative protein to evaluate the interaction behavior of oil-derived bioactive compounds in such conditions.

The fluorescence analysis (Table 3) demonstrated clear differences in the interaction of human serum albumin (HSA) with the tested systems. Native HSA showed strong intrinsic fluorescence at both characteristic peaks (Peak a: 746.30; Peak b: 854.34). The addition of Tween/NaCl caused a moderate decrease in fluorescence intensity, indicating limited nonspecific interaction.

In contrast, both amaranth oil (AMO) and the amaranth–rose formulation (AMOR) produced a markedly stronger fluorescence quenching effect, suggesting significant binding interactions between oil-derived bioactive compounds and HSA. The decrease in fluorescence intensity was greater for AMOR than for AMO at both peaks. For Peak a, the intensity decreased to 361.94 (HSA + AMO) and further to 279.37 (HAS + AMOR). A similar trend was observed for Peak b. Additionally, spectral shifts’ maxima were observed, particularly in the AMOR system, indicating conformational or microenvironmental changes in the protein structure upon ligand binding. The calculated binding properties (BPs) were higher for AMOR (37.1) compared with AMO (18.6), confirming its stronger overall interaction with albumin.

While several reports describe the interaction of plant essential oil nano emulsions with serum proteins [27], there is currently very limited information regarding the direct binding behavior of edible oils and fixed plant oils with HSA. To the best of our knowledge, systematic fluorescence-based analyses of HSA interactions with amaranth oil formulations have not been previously reported. Therefore, our results represent one of the first experimental datasets addressing albumin binding of amaranth-derived oil systems. Our fluorescence quenching and 3D spectral analyses demonstrated that both AMO and AMOR interact with HSA, but AMOR exhibited markedly stronger binding parameters and greater fluorescence attenuation. This suggests a higher affinity of AMOR-derived bioactive constituents toward albumin binding sites and indicates more pronounced modification of the protein microenvironment. From a biological standpoint, stronger albumin association may translate into improved stabilization and transport of lipophilic antioxidants such as squalene and phenolic components, potentially prolonging their local availability in tissue fluids after topical application [27].

At the same time, protein-binding strength must be interpreted carefully in the context of biosafety, since nano–bio interface interactions can influence protein conformation and downstream biological responses. Further mechanistic and structural studies are, therefore, warranted to characterize the conformational effects of AMO and AMOR binding on albumin and other extracellular proteins.

## 3. Materials and Methods

### 3.1. Materials

Amaranth (*Amaranthus cruentus* L.) seeds were obtained in 2025 from a regional supplier in Morelos, Mexico. The seeds were mechanically ground to obtain a fine particulate material prior to extraction. Oil was isolated from 100 g portions of the ground material using n-hexane extraction in a Soxhlet system operated at 60 °C for 4 h. Each extraction was performed in triplicate to ensure reproducibility. After completion of the extraction cycle, the solvent was removed under reduced pressure using a rotary evaporator R-100 with a B-100 heating bath and recirculating chiller F-100 (Büchi Labortechnik AG, Flawil, Switzerland), and the recovered oil was protected from light and stored at −20 °C until further analysis. This oil was designated as AMO (pure amaranth oil). A comparative formulation amaranth oil (*A. cruentus* L.) was enriched with rose oil from the commercially available *Rosa damascena* flower of Bulgarian origin, a material characterized by a high content of phenylethyl alcohol together with geraniol, citronellol, and nerol as major volatile constituents. This preparation was denoted as AMOR (amaranth oil with rose oil). Both AMO and AMOR samples were stored in airtight, light-protected containers at −20 °C prior to chemical and biological testing.

To obtain polar and nonpolar fractions from amaranth oils for further studies, a phase splitting procedure was applied [28]. Each oil sample was combined with Polysorbate 80 (Tween 80, Sigma-Aldrich, Saint Louis, MO, USA) at a concentration of approximately 20–25%, followed by dilution with isotonic saline (0.9% NaCl). The components were mixed in an oil/Tween 80/saline proportion of 1:1:10 (*v*/*v*/*v*). The mixtures were intensively homogenized using vortex agitation (Thermo—Shaker TS100, BioSan, Riga, Latvia) to form a temporary emulsion system. To promote phase resolution, samples were subsequently centrifuged at 5000–6000 rpm for approximately 15–20 min (MPW-351R, Warsaw, Poland). After centrifugation, two distinct layers were obtained. The upper layer represented the lipid-dominant fraction enriched in hydrophobic constituents such as triacylglycerols, sterols, and other fat-soluble compounds. The lower aqueous layer contained more polar constituents, including amphiphilic molecules and water-compatible antioxidant components. Because Tween 80 stabilizes dispersed oil droplets, mechanical separation by centrifugation and, when necessary, increased ionic strength were used to weaken the interfacial film and improve layer separation.

### 3.2. GC–MS Analysis of Fatty Acid Composition and Squalene in Amaranth Oils

The fatty acid composition and squalene content of amaranth oil (AMO) and rose-enriched amaranth oil (AMOR) were analyzed by gas chromatography coupled with mass spectrometry (GC–MS). Measurements were performed using an Agilent (Santa Clara, CA, USA) 7890A gas chromatograph fitted with an automatic sample injector (G4513A) and connected to a 5975C VL mass selective detector with a triple-axis detector system (Agilent Technologies, Inc. Wilmington, DE, USA). Chromatographic separation was carried out on an HP-DB-WAX capillary column (30 m × 0.32 mm internal diameter, 0.25 µm film thickness) produced by Agilent Technologies, Inc., Santa Clara, CA, USA. Helium served as the carrier gas at a constant flow rate of 1 mL/min. The temperature program started at 60 °C with a short initial hold, followed by a linear increase of 10 °C per minute up to 255 °C, where the temperature was maintained to ensure full elution of late-retaining components. Samples were introduced at an injection volume of 2 µL, and spectra were acquired in full scan mode. The ion source and quadrupole temperatures were maintained at 230 °C and 150 °C, respectively. Mass spectra were recorded over an *m*/*z* range of 40–1000 in positive ion mode under identical detector settings for all runs. Quantitative determination of squalene was based on an external calibration procedure using a certified analytical standard (≥99% purity, Sigma-Aldrich). The calibration curve covered nine concentration levels in the range of 0.1–15.0 mg/mL. Each sample was analyzed in triplicate. Compound identification and data processing were performed using MSD ChemStation software, v E.02.02 (Agilent Technologies, Inc., Santa Clara, CA, USA) together with the NIST MS software v 2.0 (Diablo Analytical, Inc., LTW Antioch, CA, USA). Fatty acid composition is expressed as the percentage of total identified fatty acids (%), whereas squalene content is reported as g per 100 g of oil.

### 3.3. The Total Phenolic Content in Amaranth Oils

The total phenolic content (TPC) of amaranth oils (AMO and AMOR), as well as their separated polar and nonpolar fractions, was determined using a colorimetric assay based on the Folin–Ciocalteu reaction. After extraction and fraction separation, phenolic compounds were transferred into a polar phase using an 80:20 (*v*/*v*) methanol–water solvent system. Aliquots of the obtained extracts were reacted with Folin–Ciocalteu reagent, followed by the addition of sodium carbonate solution and dilution with distilled water. The reaction mixtures were protected from light and allowed to develop color during extended incubation. Absorbance was then recorded at 765 nm using a UV–VIS spectrophotometer (Jasco V-530, Tokyo, Japan). TPC was calculated from a calibration curve prepared with gallic acid and is expressed as µg gallic acid equivalent (GAE) per mL of oil sample. Each measurement was performed in triplicate using independently prepared samples.

### 3.4. Fourier Transform Infrared Spectroscopy of Amaranth Oils

FTIR spectra of amaranth oils (AMO and AMOR) and *Rosa damascena* essential oil were recorded using an IRAffinity-1S spectrometer (Shimadzu, Tokyo, Japan) equipped with an attenuated total reflectance (ATR) accessory. Measurements were carried out in absorbance mode over the mid-infrared range of 4000–500 cm^−1^. For each sample, 120 scans were collected at a spectral resolution of 4 cm^−1^. Spectral acquisition and processing were performed using LabSolutions IR FTIR software (Shimadzu, Tokyo, Japan) (https://www.shimadzu.com/an/products/molecular-spectroscopy/ftir/ftir-spectroscopy-software/labsolutions-ir/index.html, accessed on 15 January 2026). Squalene was analyzed under the same conditions and used as a reference compound for spectral comparison.

### 3.5. Cytotoxic Activity of Amaranth Oils

The cytotoxic effects of amaranth oils, AMO and AMOR, were evaluated using an in vitro skin cell model that included two human melanoma cell lines (A375; HTB140) and non-malignant human immortalized keratinocytes (HaCaT). Cells were maintained under controlled culture conditions (37 °C, humidified atmosphere, 5% CO_2_) in growth media appropriate for each cell type [2]. All cell lines and culture media were purchased from Merck (Darmstadt, Germany). Test samples were prepared as fresh stock solutions in DMSO (10 mg/mL) and subsequently diluted with culture medium to obtain final concentrations ranging from 2.5 to 100 µg/mL. The final solvent concentration in wells did not affect cell viability. For the assay, cells were plated in 96-well plates at a density of 1.5 × 10^4^ cells per well and allowed to attach for 24 h. The medium was then replaced with treatment medium containing AMO or AMOR at the indicated concentrations. Following 24 h exposure, cytotoxicity was quantified using a lactate dehydrogenase (LDH) release assay performed according to the manufacturer’s protocol and previously validated procedures. Absorbance was recorded at 490 nm using a microplate reader (BioTek Synergy, BioTek Instruments, Winooski, VT, USA). Each experimental condition was tested in triplicate. The results are expressed as percentage cell viability relative to untreated controls (mean ± SD), and IC_50_ values were calculated from dose–response curves. Doxorubicin served as a positive cytotoxic control.

### 3.6. Evaluation of Anti-Inflammatory Activity of Amaranth Oils in Different Models

Inflammation model No 1

The first model of inflammation was based on the RAW 264.7 murine macrophages, which were seeded onto 96-well plates (1.5 × 10^5^ cells/well) and pre-treated with the amaranth oils, AMO and AMOR, in a concentration range of 5–20 µg/mL for 1 h, followed by the addition of 10 ng/mL of LPS to induce the inflammation process, according to our previous work and Paśko et al. [29]. Dexamethasone (0.5 µg/mL) was used as a reference drug. The incubation continued for the next 24 h. Cell culture supernatants were used for further analysis.

#### 3.6.1. Nitric Oxide Determination

The Griess reagent kit was obtained from Promega Corporation (Madison, Winooski, VT, USA), and a nitric oxide evaluation was performed according to the manufacturer’s instructions. The analysis was performed in RAW 264.7 cell culture supernatants after AMO and AMOR treatment on three independent replicates using Agilent BioTek Synergy H1 (Agilent Technologies, Inc., Santa Clara, CA, USA). The results are shown as % of LPS control.

#### 3.6.2. TNF-α and IL-6 Determination

According to the manufacturer’s instructions, cytokine determination was performed with rat ELISA kits (Bioassay Technology Laboratory, Shanghai, China). RAW 264.7 cell culture supernatants were used for the analysis after ingestion of amaranth oil samples (AMO and AMOR), performed for three independent replicates using a microplate reader Agilent BioTek Synergy H1 (Agilent Technologies, Inc., Santa Clara, CA, USA); the results are shown as % of LPS-treated cells.

Inflammation model No 2

Keratinocytes (HaCaT) were seeded onto 96-multi-well plates (1.5 × 10^5^ cells/well) and incubated for 24 h. To induce inflammation, the cells were treated with the cytokine cocktail, consisting of interferon γ (IFNγ) (10 ng/mL) and TNFα (20 ng/mL), and then the examined oils’ samples were added at the dose of 100 µg/mL. Dexamethasone (DEX) (0.5 µg/mL) was used as a positive control. After 48 h of incubation, the cell culture supernatants were collected for analysis.

#### 3.6.3. IL-1β, IL-6 and IL-10 Determination

According to the manufacturer’s instructions, cytokine determination was performed using human ELISA kits (Bioassay Technology Laboratory, Shanghai, China). HaCaT cell culture supernatants were used for the analysis after exposure to amaranth oil samples (AMO and AMOR), performed for three independent replicates using a microplate reader Agilent BioTek Synergy H1 (Agilent Technologies, Inc., Santa Clara, CA, USA); the results are shown as % of IFNγ+TNF-α-treated cells.

### 3.7. Fluorometric Measurements—Interaction of Amaranth Oils with Human Serum Albumin

Fluorescence spectroscopy was applied to evaluate the interaction of amaranth oil (AMO) and amaranth oil enriched with rose oil (AMOR) with human serum albumin (HSA). Measurements were performed using an FP-6500 spectrofluorometer (Jasco, Tokyo, Japan) equipped with a thermostatically controlled cell holder and a 1.0 cm quartz cuvette. Oil dispersions were prepared by mixing 250 µL of oil with a drop of Tween 80 as a solubilizing agent and diluting to 4.5 mL with 0.9% NaCl solution to obtain a stable emulsion. For fluorescence measurements, 50 µL of the prepared oil dispersion was added to the HSA solution. The protein solution consisted of 2.0 × 10^−5^ mol/L HSA prepared in 0.05 mol/L Tris–HCl buffer containing 0.1 mol/L NaCl (pH 7.4). Samples were equilibrated prior to measurement. Two-dimensional fluorescence (2D-FL) emission spectra were recorded in the range of 310–500 nm with excitation fixed at 295 nm. Three-dimensional fluorescence (3D-FL) spectra were obtained by scanning emission wavelengths from 200 to 550 nm while varying excitation wavelengths from 200 to 500 nm. Binding interactions were assessed by analyzing changes in fluorescence intensity at characteristic HSA peaks (Peak a and Peak b) after the addition of AMO or AMOR, relative to native HSA. The percentage decrease in fluorescence intensity and spectral shifts were used as indicators of ligand–protein interaction strength and microenvironmental changes around aromatic amino acid residues. Reference antioxidant standards (gallic acid) were measured under analogous conditions to support spectral interpretation.

### 3.8. Statistical Approach

All experiments were performed in triplicate, and the data are reported as the mean ± standard deviation (SD). The results obtained for pseudocereal oils were analyzed using a one-way analysis of variance (ANOVA) followed by a post hoc Tukey’s test using STATISTICA v. 13.3. (TIBCO Software Inc., Palo Alto, CA, USA). The differences among the groups were considered statistically significant when the *p*-values were 0.05 or less.

## 4. Conclusions

The stronger biological activity of the AMOR formulation, both in terms of anti-inflammatory efficacy and selective cytotoxicity, appears to be associated with its enriched composition, particularly the higher content of polyphenolic compounds derived from rose oil enrichment, as well as the generally high level of squalene. This interpretation is further supported by our fluorescence binding studies, which demonstrated markedly stronger interactions between AMOR and human serum albumin. Enhanced albumin-binding capacity may improve the local retention of bioactive lipophilic constituents, thereby prolonging their biological effects after topical application. Together, these results highlight the added value of combining pseudocereal oils with essential oils of known bioactivity to create multifunctional formulations with both preventive and therapeutic dermatological potential. However, our study also supports the idea of topical use of AMO, despite its weaker effects, as compared to AMOR, and provides convincing arguments for the continuation of such research.

## Figures and Tables

**Table 1:** Fatty acid profile, squalene and total phenolic content of amaranth oil (AMO) and amaranth oil with rose oil (AMOR).

Parameters	AMO	AMOR
**Fatty acid profile [%]**		
Palmitic acid C 16:0	4.5 ± 0.1 *	11.1 ± 1.2 *
Stearic acid C 18:0	8.1 ± 0.5	5.9 ± 0.8
Oleic acid C 18:1 n-9	27.6 ± 3.2	31.1 ± 1.4
Linoleic acid C 18:2 n-6	46.5 ± 5.1	48.9 ± 4.1
**Squalene [g/100 g]**	6.9 ± 0.9	7.9 ± 1.1
**Total polyphenols [µg GAE/mL]**		
Amaranth oil	113.9 ± 1.2 ***	252.7 ± 4.9 ***
Lipophilic fraction of oil	36.6 ± 2.2 ***	123.9 ± 2.0 ***
Hydrophilic fraction of oil	77.5 ± 1.7 **	128.8 ± 3.2 **

* *p* < 0.05; ** *p* < 0.01; *** *p* < 0.001.

**Table 2:** Cytotoxic activity of amaranth oils and doxorubicin (reference drug) to non-cancerous (HaCaT) and cancer (HTB140, A375) skin cells after 24 h incubation expressed as IC_50_ values (µg/mL).

Type of Cells	AMO	AMOR	Doxorubicin
HaCaT	>C_max_	>C_max_	3.20
HTB140	27.9	18.9	4.45
A375	14.4	3.8	0.37

AMO—amaranth oil; AMOR—rose-enriched amaranth oil.

**Table 3:** Fluorescence results of 3D measurements of amaranth oils in interaction with human serum albumin (HSA).

Sample	Peak a		Peak b		BP
	λ_ex_/λ_em_ (nm/nm)	Int F_0_	λ_ex_/λ_em_ (nm/nm)	Int F_0_	%
HSA	228/347	746.30	280/350	854.34	
HSA + Tween NaCl	227/347	444.43	280/355	822.00	
HSA + AMO	228/357	361.94	280/357	797.90	18.6
HSA + AMOR	232/358	279.37	284/359	739.70	37.1

Int F_0_—fluorescence intensity. BP—binding properties.

## Data Availability

Data will be made available on request.

## Abbreviations

The following abbreviations are used in this manuscript:

A375	malignant melanoma cell line
AMO	amaranth oil
AMOR	amaranth oil enriched with rose oil
BP	binding parameter
COX-2	cyclooxygenase-2
FTIR	Fourier transform infrared spectroscopy
GAE	gallic acid equivalents
HaCaT	human adult low calcium temperature keratinocytes
HSA	human serum albumin
HTB140	metastatic melanoma cell line
IC_50_	half maximal inhibitory concentration
IL-1β	interleukin-1 beta
IL-6	interleukin-6
IL-10	interleukin-10
LPS	Lipopolysaccharide
MAPK	mitogen-activated protein kinase
NF-κB	nuclear factor kappa-light-chain-enhancer of activated B cells
NO	nitric oxide
Nrf2	nuclear factor erythroid 2–related factor 2
PBS	phosphate-buffered saline
ROS	reactive oxygen species
SOD	superoxide dismutase
TNF-α	tumor necrosis factor alpha
UVB	ultraviolet B radiation
3D-FL	three-dimensional fluorescence spectroscopy
